# A Detailed Analysis of the Dynamic Behavior of a MEMS Vibrating Internal Ring Gyroscope

**DOI:** 10.3390/mi15091107

**Published:** 2024-08-30

**Authors:** Waqas Amin Gill, Ian Howard, Ilyas Mazhar, Kristoffer McKee

**Affiliations:** Department of Mechanical Engineering, Curtin University, Perth, WA 6845, Australia; i.howard@exchange.curtin.edu.au (I.H.); i.mazhar@curtin.edu.au (I.M.)

**Keywords:** MEMS, MEMS gyroscope, vibrating ring gyroscope, dynamics, motion equations, resonance frequency, ring resonator, inertial sensor

## Abstract

This paper presents the development of an analytical model of an internal vibrating ring gyroscope in a Microelectromechanical System (MEMS). The internal ring structure consists of eight semicircular beams that are attached to the externally placed anchors. This research work analyzes the vibrating ring gyroscope’s in-plane displacement behavior and the resulting elliptical vibrational modes. The elliptical vibrational modes appear as pairs with the same resonance frequency due to the symmetric structure of the design. The analysis commences by conceptualizing the ring as a geometric structure with a circular shape possessing specific dimensions such as thickness, height, and radius. We construct a linear model that characterizes the vibrational dynamics of the internal vibrating ring. The analysis develops a comprehensive mathematical formulation for the radial and tangential displacements in local polar coordinates by considering the inextensional displacement of the ring structure. By utilizing the derived motion equations, we highlight the underlying relationships driving the vibrational characteristics of the MEMS’ vibrating ring gyroscope. These dynamic vibrational relationships are essential in enabling the vibrating ring gyroscope’s future utilization in accurate navigation and motion sensing technologies.

## 1. Introduction

Microelectromechanical systems’ (MEMSs) inertial sensors have become integral to many modern-day smart devices because of their micro size, energy efficiency, low cost, and high-performance characteristics [1,2,3,4,5,6,7]. Among the various MEMS inertial sensors, vibrating ring gyroscopes stand out as imminent inertial sensors that are extensively used in many applications to measure and control the position of the system. Their usage has increased tremendously in smart electronics, automotive, military, biomedical, and space applications [8,9,10,11,12,13,14,15,16,17].

The world’s demand for the downscaling of these microscale inertial sensors with improved performance is increasing. For the development of high-performance devices, it is important to understand the dynamic behaviors of these sensors in different environments. Most MEMSs’ vibrating gyroscopes operate on the translational motion principle of a single-proof mass system. Because of the single-proof mass system, translational motion delivers excellent gyroscopic performance with simple microfabrication processes. While some gyroscopes operate on the rotational motion of the spring-mass system, these systems require complex operational mechanisms and microfabrication processes.

**Figure 1:**
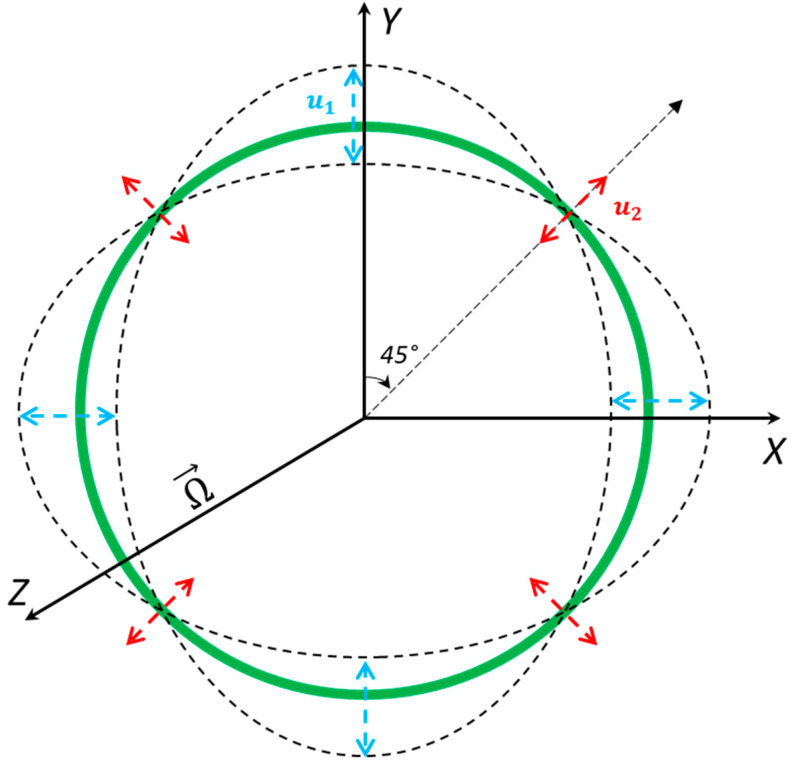
The dynamic illustration of the primary oscillation of the vibrating ring gyroscope [22].

MEMS vibrating ring gyroscopes, like other vibrating gyroscopes, operate on the fundamentals of the Coriolis effect [14,18,19,20,21]. The vibrating ring structure is set to continuously oscillate in the X and Y axes. These oscillations can be seen as elliptical shapes in the X and Y axes. The primary oscillation has four nodes where the vibrating ring has no displacement; these four nodes are located at 45 degrees between both axes. The vibrating ring’s primary oscillation dynamic system is shown in Figure 1. When the vibrating ring structure is exposed to the external rotation along the Z-axis (in this case), the secondary oscillation starts appearing in the sensing direction at 45 degrees between the X and Y axes due to the Coriolis force.

## 2. Dynamics of MEMS Vibrating Ring Gyroscope

The fundamental understanding of the dynamics of MEMSs’ vibrating ring gyroscopes starts with a single vibrating ring mass system placed above the substrate. The ring structure is attached with flexible suspension beams and a centrally placed anchor support. A linear vibrating ring gyroscope is required to oscillate in two adjacent axes with identical vibrational modes for the gyroscope’s operation. Suppose the vibrating ring gyroscope drives along the X and Y axes with the displacement $u_{1}$, and when the gyroscope experiences rotation along the Z axis, the displacement $u_{2}\;$ appears at 45 degrees between the X and Y axes, as shown in Figure 1. The dynamic system of a vibrating ring gyroscope is referred to as having two degrees of freedom.

**Figure 2:**
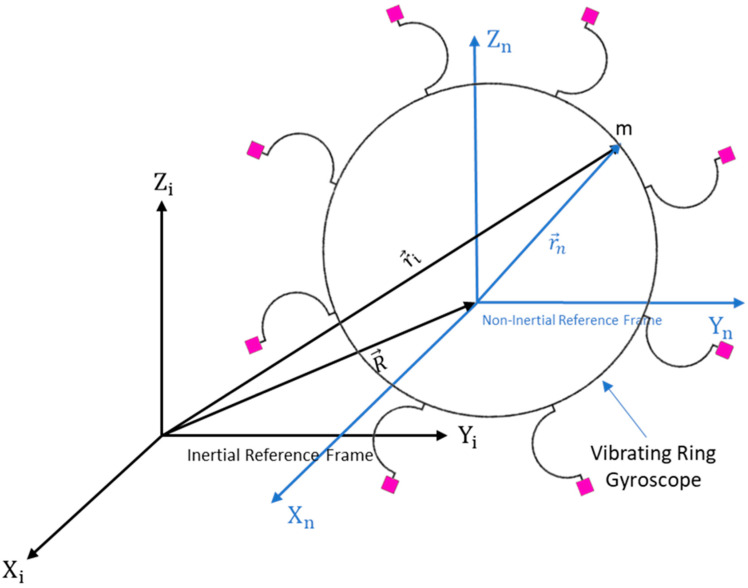
A schematic representation of inertial and non-inertial reference frames with position vectors.

The first and foremost task in analyzing the MEMS vibrating ring gyroscope’s dynamic behavior is to study the effects of various forces on the gyroscope. In particular, the influence of the rotational-induced Coriolis force on the body in the inertial system and observations from a rotating reference frame must be studied. To determine the equations of the forces acting on the system, it is necessary to examine the motion of an object in a rotating reference frame relative to an inertial reference frame. The inertial and non-inertial reference frames are explained in detail below and shown in Figure 2.

### 2.1. Reference Frames

Assuming a non-inertial rotating reference frame with constant acceleration relating to the inertial reference frame, we can start with Newton’s second law of motion and determine the inertial force. The inertial reference frame and non-inertial reference frame with a point mass “*m*” and position vectors are shown in Figure 2. Descriptions of the elements are written below:

$I_{i}$ = the inertial reference frame (stationary);
$I_{n}$ = the non-inertial reference frame (accelerating);
$\vec{R}$ = the position vector of non-inertial reference frame ”$I_{n}$” with respect to the inertial frame “$I_{i}$”;
$\dot{\vec{R}}$ = the velocity of the non-inertial reference frame “$I_{n}$” with respect to “$I_{i}$”;
$\ddot{\vec{R}}$ = the acceleration of the non-inertial frame “$I_{n}$” with respect to “$I_{i}$”;
$\vec{r_{i}}$ = the position vector relative to the inertial reference frame;
$\vec{r_{n}}$ = the position vector relative to the non-inertial reference frame;
$\ddot{\vec{r_{i}}}$ = the acceleration of the inertial reference frame;
$\ddot{\vec{r_{n}}}$ = the acceleration of the non-inertial reference frame.

An observer from an inertial reference frame (stationary) can see a point mass “*m*” with a position vector ${\vec{r}}_{i}$, and hence, the force acting on the inertial reference frame is given as Equation (1).

(1) $$\vec{F}=m\ddot{{\vec{r}}_{i}}$$

We start with the position vectors and then take the second derivative of the position vectors.

(2) $${\vec{r}}_{i}=\vec{R}+{\vec{r}}_{n}$$

(3) $$\ddot{{\vec{r}}_{i}}=\ddot{\vec{R}}+\ddot{{\vec{r}}_{n}}$$

We then substitute Equation (3) into Equation (1).

(4) $$\vec{F}=m(\ddot{\vec{R}}+\ddot{{\vec{r}}_{n}})$$

(5) $$\vec{F}-m\ddot{\vec{R}}=m\ddot{{\vec{r}}_{n}}$$

(6) $$\vec{F}-\vec{F_{o}}=\vec{F_{n}}$$

The force $\vec{F_{o}}$ is the inertial force observed from the non-inertial reference frame, $\vec{F}$ is the force which is observed from the inertial reference frame, and $\vec{F_{n}}$ is the force from the non-inertial reference frame.

Let us consider a vector P that interacts with the time derivative of the inertial reference frame to the time derivative of the non-inertial reference frame. The vector, P, will rotate with angular velocity in a non-inertial reference frame with respect to the inertial reference frame and it is represented as Equation (7).

(7) $${\left(\frac{d}{dt}\vec{P}\right)}_{i}={\left(\frac{d}{dt}\vec{P}\right)}_{n}+\vec{\Omega }\times \vec{P}$$

Furthermore, we investigate the equation relating to the velocities in the inertial and non-inertial reference frames by using Equation (7) and considering the position vector $\vec{P}=\vec{r}$. The next step is to investigate Equation (7), which relates to the accelerations present in the inertial and non-inertial reference frames.

(8) $${\left(\frac{d}{dt}\right)}_{i}{\left(\frac{d\vec{r}}{dt}\right)}_{i}={\left(\frac{d}{dt}\right)}_{n}{\left(\frac{d\vec{r}}{dt}\right)}_{i}+\vec{\Omega }\times {\left(\frac{d\vec{r}}{dt}\right)}_{i}$$

As given in Equation (7), the velocity observed by the inertial reference frame is equal to the velocity and angular velocity experienced by the non-inertial reference frame; it is ${\left(\frac{d}{dt}\vec{r}\right)}_{i}={\left(\frac{d}{dt}\vec{r}\right)}_{n}+\vec{\Omega }\times \vec{r}$ and substituted into Equation (8).

(9) $${\left(\frac{d^{2}\vec{r}}{dt^{2}}\right)}_{i}={\left(\frac{d}{dt}\right)}_{n}\left[{\left(\frac{d\vec{r}}{dt}\right)}_{n}+\vec{\Omega }\times {\vec{r}}_{n}\right]+\vec{\Omega }\times \left[{\left(\frac{d\vec{r}}{dt}\right)}_{n}+\vec{\Omega }\times {\vec{r}}_{n}\right]$$

(10) $${\left(\frac{d^{2}\vec{r}}{dt^{2}}\right)}_{i}={\left(\frac{d^{2}\vec{r}}{dt^{2}}\right)}_{n}+{\left(\frac{d}{dt}\right)}_{n}\left(\vec{\Omega }\times {\vec{r}}_{n}\right)+\vec{\Omega }\times {\left(\frac{d\vec{r}}{dt}\right)}_{n}+\vec{\Omega }\times \left(\vec{\Omega }\times {\vec{r}}_{n}\right)$$

(11) $${\left(\frac{d^{2}\vec{r}}{dt^{2}}\right)}_{i}={\left(\frac{d^{2}\vec{r}}{dt^{2}}\right)}_{n}+2\vec{\Omega }\times {\left(\frac{d\vec{r}}{dt}\right)}_{n}+\vec{\Omega }\times \left(\vec{\Omega }\times {\vec{r}}_{n}\right)$$

Acceleration can be observed in the inertial reference frame and is equal to the acceleration in the non-inertial reference frame with Coriolis acceleration and centripetal acceleration. 

**Figure 3:**
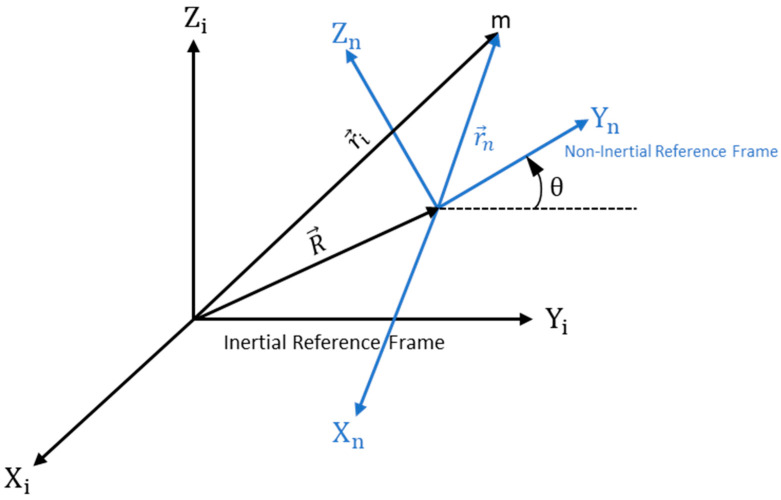
A schematic view of the position vector of a non-inertial reference frame with respect to an inertial reference frame with rotation applied.

Now consider the non-inertial reference frame with the inertial reference frame. A rotation “θ” is applied into the non-inertial reference frame and represented in Figure 3.

(12) $${\vec{r}}_{i}=\vec{R}+{\vec{r}}_{n}$$

(13) $${\left(\frac{d\vec{r}}{dt}\right)}_{i}=\left(\frac{d\vec{R}}{dt}\right){+\left(\frac{d\vec{r}}{dt}\right)}_{n}+\left(\frac{d\vec{\theta }}{dt}\times \vec{r_{n}}\right)$$

(14) $$\begin{array}{c} {\left(\frac{d^{2}\vec{r}}{dt^{2}}\right)}_{i}=\left(\frac{d^{2}\vec{R}}{dt^{2}}\right)+{\left(\frac{d^{2}\vec{r}}{dt^{2}}\right)}_{n}+{\left(\frac{d\theta }{dt}\right)}_{n}{\times \left(\frac{d\vec{r}}{dt}\right)}_{n}+{\left(\frac{d\theta }{dt}\right)}_{n}\times \left(\frac{d\vec{\theta }}{dt}\times \vec{r_{n}}\right)\\ +\left(\frac{d^{2}\theta }{dt^{2}}\right)\times \vec{r_{n}}+{\left(\frac{d\theta }{dt}\right)}_{n}{\times \left(\frac{d\vec{r}}{dt}\right)}_{n} \end{array}$$

(15) $$\begin{array}{c} {\left(\frac{d^{2}\vec{r}}{dt^{2}}\right)}_{i}={\left(\frac{d^{2}\vec{R}}{dt^{2}}\right)+\left(\frac{d^{2}\vec{r}}{dt^{2}}\right)}_{n}+\left(\frac{d\vec{\Omega }}{dt}\right)\times \vec{r_{n}}+2\vec{\Omega }\times {\left(\frac{d\vec{r}}{dt}\right)}_{n}\\ +\vec{\Omega }\times \left(\vec{\Omega }\times {\vec{r}}_{n}\right) \end{array}$$

(16) $$\ddot{{\vec{r}}_{i}}=\ddot{\vec{R}}+\ddot{{\vec{r}}_{n}}+\dot{\vec{\Omega }}\times \vec{r_{n}}+2\vec{\Omega }\times \dot{{\vec{r}}_{n}}+\vec{\Omega }\times \left(\vec{\Omega }\times {\vec{r}}_{n}\right)$$

The concept of the dynamics of the gyroscope when we apply Newton’s second law of motion equation on the acceleration with the gyroscope’s mass is understood as written in Equations (17) and (18).

(17) $$F=m\ddot{{\vec{r}}_{i}}$$

(18) $$F_{A}=m\left[\ddot{\vec{R}}+\ddot{{\vec{r}}_{n}}+\dot{\vec{\Omega }}\times \vec{r_{n}}+2\vec{\Omega }\times \dot{{\vec{r}}_{n}}+\vec{\Omega }\times \left(\vec{\Omega }\times {\vec{r}}_{n}\right)\right]$$

Here, $F_{A}$ is the applied force on the reference frame, $\ddot{{\vec{r}}_{i}}$ is the acceleration experienced by the inertial reference frame, the combination of ($\ddot{\vec{R}}+\ddot{{\vec{r}}_{n}}+\dot{\vec{\Omega }}\times \vec{r_{n}}$) is the acceleration observed by the non-inertial reference frame, $\ddot{\vec{R}}$ is the linear acceleration observed in the non-inertial reference frame,$\;\ddot{{\vec{r}}_{n}}$ is the acceleration in the non-inertial reference frame, and $\vec{\Omega }$ represents the non-inertial reference frame experiencing angular velocity. The expression $\vec{\Omega }\times \left(\vec{\Omega }\times {\vec{r}}_{n}\right)$ represents the centripetal acceleration, and the expression $2\vec{\Omega }\times \dot{{\vec{r}}_{n}}$ represents the Coriolis acceleration. The Coriolis acceleration is the main concept that transfers the rotational energy of the non-inertial reference frame for measuring the rotation rate with respect to the inertial reference frame. 

### 2.2. Motion Equations of Vibrating Ring Gyroscope

The MEMS vibrating ring gyroscope is an inertial sensor that measures and controls precise and accurate angular rates in dynamic motion environments. Utilizing the Coriolis force and resonant frequency principles, this miniaturized device offers excellent sensitivity and stability, making it ideal for applications such as navigation, stabilization, and motion tracking. Dual-axis gyroscopes, which measure angular motion in two orthogonal axes, tend to be larger and consume more power than the MEMS vibrating ring gyroscope. The MEMS vibrating ring gyroscope performs better than dual-axis and tuning fork gyroscopes in several key aspects, including size, power consumption, sensitivity, and reliability. Its symmetrical design and operating principles make it a highly attractive sensor for various applications, including navigation, stabilization, and motion tracking [23,24,25].

In this section, we present the development of a linear mechanical model for a vibrating ring resonator. The ring structure consists of eight semicircular support springs that are connected with a fixed anchor support [26,27,28,29,30]. The vibrational mode shapes characterize the oscillations of the ring. In the case of symmetric design structures, such as a vibrating ring, it is observed that mode shapes appear in pairs comprising identical resonance frequencies and being oriented orthogonally to each other.

#### 2.2.1. Coordinates for Vibrating Ring Gyroscope

**Figure 4:**
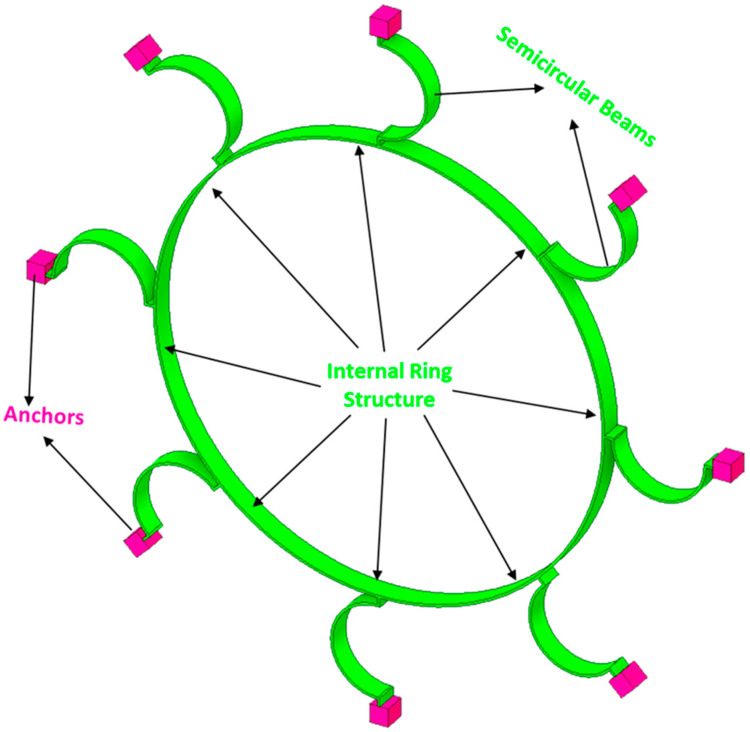
A schematic view of the internal vibrating ring gyroscope.

A proposed MEMS vibrating internal ring resonator with eight semicircular beams connected to externally placed anchors makes a complete vibrating ring gyroscope, as shown in Figure 4. In this study, we will consider the in-plane displacements of the vibrating ring and evaluate the elliptical vibrational modes. A vibrating ring gyroscope possesses a symmetric design structure, so the vibrational modes occur in identical pairs with the same resonance frequency.

**Figure 5:**
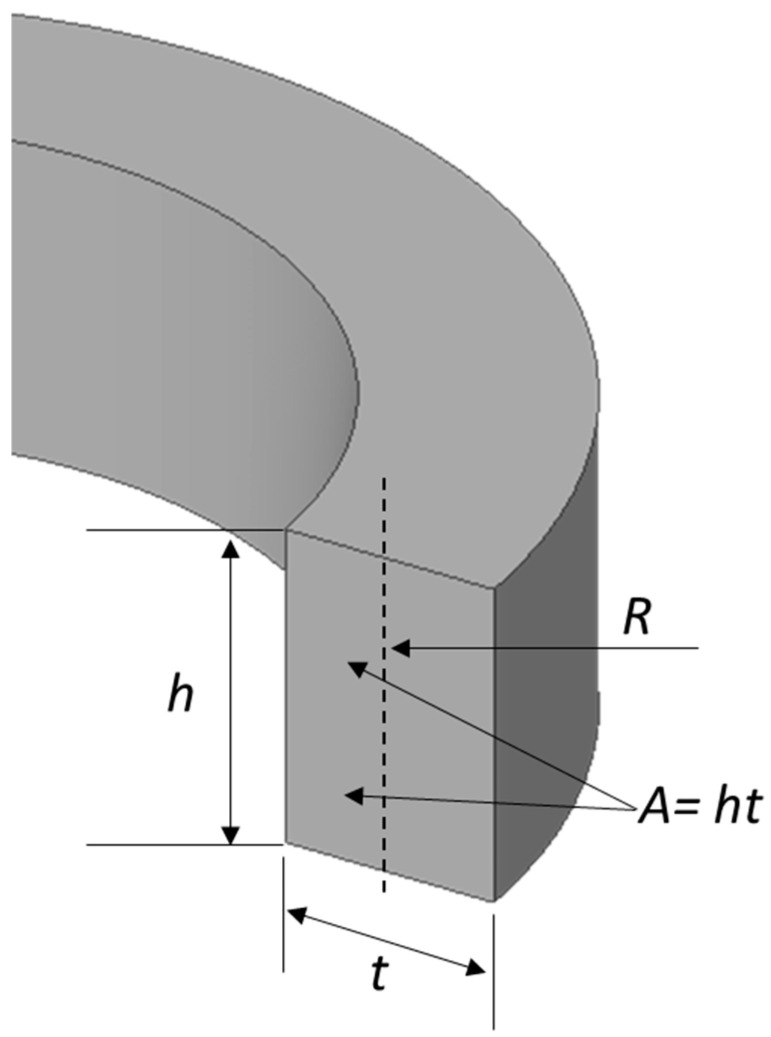
A cross-sectional area “*A*” of the ring structure with the centre line radius “*R*”, height “*h*”, and thickness “*t*”.

Firstly, the vibrating ring is considered as a perfect round ring with thickness “*t*”. The cross-sectional area of the ring is shown in Figure 5, where “*h*” is the height, *R* is the radius of the ring center line, “*t*” is the thickness of the ring, and “*A*” is the cross-sectional area of the ring. The ring’s displacement and elastic deformation are relatively small, so it is considered as a linear model of the vibrating ring gyroscope.

**Figure 6:**
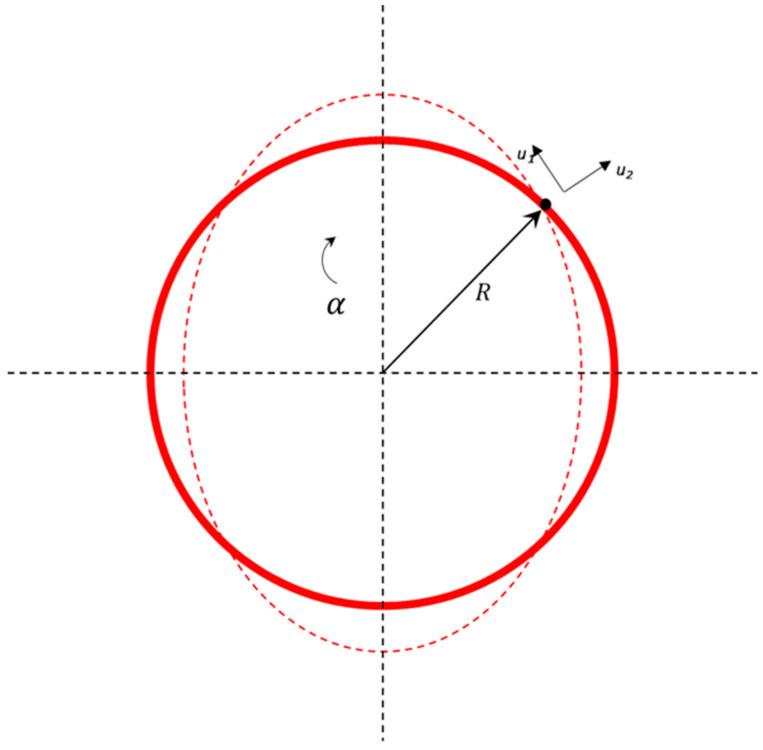
The displacement of the in-plane ring as an elliptical mode of vibration.

The analysis starts with the ring motion and considers the elliptical vibrational mode only, which is also represented by 2θ. The in-plane vibrations of a vibrating ring are shown in Figure 6. Each infinitesimal element in-plane vibration possesses two degrees of freedom. The displacements can be characterized in local polar coordinates using the variables $u_{1}$ and $u_{2}$ under the assumption that inextensional displacement is present in the ring. The radial displacement $u_{1}$ and tangential displacement $u_{2}$ of the ring in relation to the mid-section of the ring at a specific ring angle α can be expressed as Equations (18) and (19). Here, $G_{1}$ and $G_{2}$ are the generalized coordinates that provide information about the amplitude of the radial and tangential displacements of the vibrating ring at a specific angular position α. $G_{1}$ and $G_{2}$ characterize the elliptical vibrational mode of the ring. These generalized coordinates are quite significant to determine the mode shape and nature of the elliptical deformation of the ring during vibrational mode.

(19) $$u_{1}\left(\alpha \right)=G_{1}\cos(2\alpha )+G_{2}\sin\left(2\alpha \right)$$

(20) $$u_{2}\left(\alpha \right)=\frac{G_{1}}{2}\sin(2\alpha )-\frac{G_{2}}{2}\sin\left(2\alpha \right)$$

#### 2.2.2. Effects of Various Energies

To understand the motion equations of the vibrating ring structure, we will evaluate the effects of various energies on the ring resonator and the elliptical mode of vibration of the ring structure. They are also considered as the coordinates of 2*θ* elliptical vibrations, as shown in Figure 6. The Lagrange equation is considered to determine the motion equation for the vibrating ring gyroscope. The given scheme of the energy equation is shown below. Here, L represents the Lagrangian function, which is presented as $L=K_{E}-(R_{E}+U_{E}+U_{SC}$), where $K_{E}$ is the kinetic energy, $R_{E}$ is the Rayleigh damping, $U_{E}$ is the strain energy, and $U_{SC}$ is the strain energy of the semicircular support beams. The mechanical system with two degrees of freedom is described by the following Lagrange’s equation of motion:

$$\frac{d}{dt}\left(\frac{\partial L}{\partial {\dot{e}}_{i}}\right)-\frac{\partial L}{\partial e_{i}}=\frac{\partial E_{i}}{\partial e_{i}}$$

(21) $$\frac{d}{dt}(\frac{\partial K_{E}}{\partial {\dot{e}}_{i}})-\frac{\partial K_{E}}{\partial e_{i}}-\frac{\partial R_{E}}{\partial {\dot{e}}_{i}}-\frac{\partial \left(U_{E}+U_{SC}\right)}{\partial e_{i}}=\frac{\partial E_{i}}{\partial e_{i}},\;\;(i=1,2,\ldots ,n)$$

##### Kinetic Energy

To find the kinetic energy of the vibrating ring, the displacements of the ring and the central anchor should be considered. The absolute displacements of the ring are denoted by $u_{1}$ and $u_{2}$, and those for the central anchor are denoted by $x_{a}$ and $y_{a}$. To determine the absolute displacements, denoted by x and y, of the vibrating ring system, the displacement terms of the vibrating ring and centrally placed anchor need to be combined. The absolute velocities $v_{1}$ and $v_{2}\;$ of the ring are given in Equations (22) and (23).

(22) $$v_{1}=\frac{dx}{dt}=\frac{du_{1}}{dt}cos\alpha +\frac{du_{2}}{dt}sin\alpha +\frac{dx_{a}}{dt}$$

(23) $$v_{2}=\frac{dy}{dt}=\frac{du_{2}}{dt}cos\alpha -\frac{du_{1}}{dt}sin\alpha +\frac{dy_{a}}{dt}$$

Hence, the kinetic energy of the vibrating ring is written as Equation (24):

(24) $$K_{E}=\frac{1}{2}A\rho R\int_{0}^{2\pi }({v_{1}}^{2}+{v_{2}}^{2})d\alpha$$

Here, ρ is the density, R is the radius, and A is the cross-sectional area of the vibrating ring gyroscope. By substituting Equations (22) and (23) into Equation (24) and then further solving it, it is found that the kinetic energy of the ring is presented as Equation (25), where m represents the mass of the vibrating ring.

(25) $$K_{E}=\frac{1}{2}m\left[\frac{5}{8}{\left(\frac{dG_{1}}{dt}\right)}^{2}+\frac{5}{8}{\left(\frac{dG_{2}}{dt}\right)}^{2}\right]$$

Equation (25) provides the detailed dynamic movement of the ring through the kinetic energy equation. The equation considers both the rigid body displacement of the ring and the elliptical mode of the ring structure, which pertain to the displacements with the in-plane of the motion of the ring. Moreover, the equation considers the influence of base excitation that refers to the external forces applied on the anchors. The important feature is the decoupling of the generalized coordinates $G_{1}$ and $G_{2}$ that represent the motion of the ring. The coordinates in the equation are not mutually dependent in calculating the kinetic energy. The phenomenon of uncoupling suggests that the various components of motion, such as in-plane vibrations and base excitation, contribute to the overall kinetic energy of the vibrating ring gyroscope system.

##### Elastic Strain Energy

In relation to the concept of the elastic strain energy, it is supposed that the vibrating ring possesses elastic properties and corresponds to Hook’s law. The law states that stress is directly proportional to strain, with constant proportionality being Young’s modulus, where “E” represents the material. Hence, the elastic strain energy “$U_{E}$” is calculated as follows:

(26) $$U_{E}=\frac{ER}{2}\int_{0}^{2\pi }\int \epsilon^{2}dAd\alpha$$

where R is the radius of the ring, and ϵ is the normal strain of the ring. The normal strain of the ring can be calculated as follows:

(27) $$\epsilon =\frac{1}{R}\left[-u_{1}+\frac{\partial u_{2}}{\partial \alpha }-\frac{x}{R}\frac{\partial }{\partial \alpha }\left(u_{2}+\frac{\partial u_{1}}{\partial \alpha }\right)\right]$$

The term “x” refers to the distance of a point of the ring from the central axis. As mentioned, the inextensionality of the ring makes ϵ=0 at x=0. Therefore, Equation (28) becomes simpler and shows the extensional strain of the ring.

(28) $$u_{1}=\frac{\partial u_{2}}{\partial \alpha }$$

We can obtain the elastic strain energy equation by putting Equations (27) and (28) into Equation (26).

(29) $$U_{E}=\frac{EI}{2R^{3}}\int_{0}^{2\pi }{\left(u_{1}+\frac{\partial^{2}u_{2}}{{\partial \alpha }^{2}}\right)}^{2}d\alpha$$

(30) $$U_{E}=\frac{EI\pi }{2R^{3}}\left(9{G_{1}}^{2}+9{G_{2}}^{2}\right)$$

The elastic strain energy equation shows the elliptical modes of the ring and shows that the generalized ring coordinates are not connected in the strain energy equation.

##### Strain Energy of Semicircular Beams

The flexible beams are the integral parts of any MEMS vibrating gyroscope system. The semicircular beams make up the structure system supporting the vibrating ring in the vibrating ring gyroscope system. In Figure 4, the eight semicircular beams are connected to support the ring structure. As we can see, the semicircular beams are relatively small compared to the ring structure. Therefore, we considered neglecting the tiny mass of the semicircular beams. In this scenario, the kinetic energy of the semicircular beam could be considered zero.

**Figure 7:**
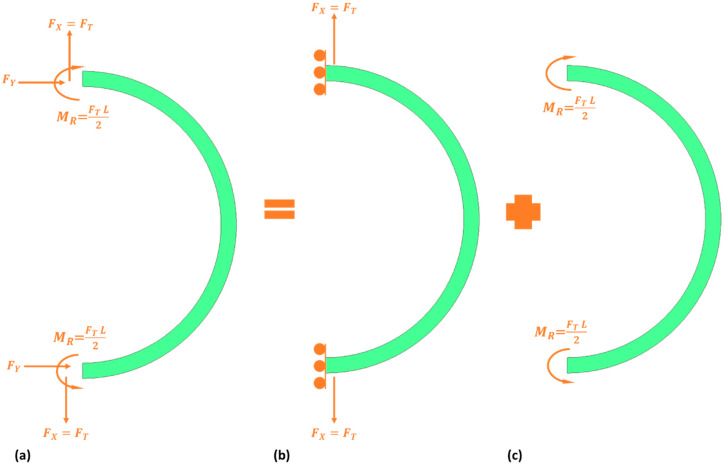
A model depicting the semicircular support stiffness: (**a**) complete; (**b**) tangential; (**c**) radial [31].

When the force is applied to the vibrating structure, the semicircular beams undergo radial and tangential directions. The semicircular beam’s radial and tangential stiffnesses are considered separately, as shown in Figure 7 [31].

The strain energy equation determines the stiffness constant for a semicircular beam when subjected to an applied force or external rotation. This results in tangential and radial displacements, respectively. Therefore, the tangential and radial stiffness constants will be further determined.

The tangential strain energy equation for tangential displacement is represented by Equation (31), where E is Young’s modulus, I is the moment of inertia, M is the bending moment experienced, and dx is the differential width of the semicircular beam.

(31) $$U_{T}=\int \frac{M^{2}}{2EI}dx$$

The above equation is further solved [31] and presented as Equation (32).

(32) $$U_{T}=\int_{0}^{\frac{\pi }{2}}\frac{1}{EI}{\left[M_{I}-\frac{F_{T}R}{2}(1-\cos\alpha )\right]}^{2}R\;d\alpha$$

where $F_{T\;}$ is the net applied force, R is the radius of the semicircular support beam, and $M_{I}$ is the imaginary moment experienced by the semicircular support beam when subjected to the external applied force. Further solving the tangential strain energy equation determines the tangential stiffness constant.

(33) $$\frac{1}{k_{T}}=\frac{2R^{3}}{EI}\int_{0}^{\frac{\pi }{2}}{\left(\frac{\cos\alpha }{2}-\frac{1}{\pi }\right)}^{2}d\alpha$$

The radial strain energy equation for radial displacement is presented as Equation (34).

(34) $$U_{T}=\int_{0}^{\frac{\pi }{2}}\frac{{M_{R}}^{2}R}{EI}dx$$

The above equation is further solved to determine the radial stiffness constant, which is shown as Equation (35), where l is the length of the beam.

(35) $$\frac{1}{k_{R}}=\frac{\pi Rl^{2}}{4EI}$$

The complete stiffness constant equation for the semicircular support beam can be written as

(36) $$\frac{1}{k_{T}}+\frac{1}{k_{R}}=\frac{2R^{3}}{EI}\int_{0}^{\frac{\pi }{2}}{\left(\frac{\cos\alpha }{2}-\frac{1}{\pi }\right)}^{2}d\alpha +\frac{\pi Rl^{2}}{4EI}$$

There are eight semicircular support beams attached to the vibrating ring. Therefore, the total strain energy $U_{SC}$ for semicircular beams could be written as Equation (37), where *i*th represents the semicircular support beam position attached to the ring.

(37) $$U_{SC}=\sum_{i=1}^{8}\frac{{u_{1}}^{2}}{2}k_{R}+\sum_{i=1}^{8}\frac{{u_{2}}^{2}}{2}k_{T}$$

By substituting Equations (19) and (21) into Equation (37), we can further determine the total strain energy $U_{SC}$ for eight semicircular beams attached to the vibrating ring.

(38) $$U_{SC}=\frac{k_{R}}{2}\left(4{G_{1}}^{2}+4{G_{2}}^{2}\right)+\frac{k_{T}}{2}\left({G_{1}}^{2}+{G_{2}}^{2}\right)$$

##### Damping Energy

The damping energy for MEMSs’ inertial sensors in the vacuum is generally referred to as thermoelastic damping [3,32]. The energy dissipation factor is considered as the Rayleigh damping function. The Rayleigh damping function for the MEMS vibrating ring gyroscope system [33] can be expressed as Equation (39).

(39) $$R_{E}=\frac{1}{2}\left[c\left({\left(\frac{dG_{1}}{dt}\right)}^{2}+{\left(\frac{dG_{2}}{dt}\right)}^{2}\right)\right]$$

The given expressions $G_{1}$ and $G_{2}$ are elliptical mode shapes, which are generally represented by 2θ.

To derive the equations of motion for the vibrating internal ring gyroscope, the Lagrange’s equation is given as

(40) $$\frac{d}{dt}\left(\frac{\partial K_{E}}{\partial {\dot{e}}_{i}}\right)-\frac{\partial K_{E}}{\partial e_{i}}-\frac{\partial R_{E}}{\partial {\dot{e}}_{i}}-\frac{\partial (U_{E}+U_{SC})}{\partial e_{i}}=\frac{{\partial E}_{i}}{\partial e_{i}}$$

The vibrating internal ring gyroscope resonator mechanical model is shown in Figure 4. The derived energy equations will be substituted into Equation (21) to find the motion equation of a vibrating ring gyroscope.

(41) $$\frac{5}{8}m\ddot{G_{1}}+c\dot{G_{1}}+(4k_{R}+k_{T}+\frac{9EI\pi }{2R^{3}})G_{1=}\dot{E}$$

(42) $$\frac{5}{8}m\ddot{G_{2}}+c\dot{G_{2}}+(4k_{R}+k_{T}+\frac{9EI\pi }{2R^{3}})G_{2=}\dot{E}$$

To simplify the equations of motion for the vibrating ring gyroscope, we consider $\frac{5}{8}m=m_{G}$, $c=c_{G}$, and $k_{G}=4k_{R}+k_{T}+\frac{9EI\pi }{2R^{3}}$.

(43) $$m_{G}\ddot{G_{1}}+c_{G}\dot{G_{1}}+k_{G}G_{1=}F_{1}$$

(44) $$m_{G}\ddot{G_{2}}+c_{G}\dot{G_{2}}+k_{G}G_{2=}F_{2}$$

The natural frequency of the gyroscope is $\omega_{G}=\sqrt{\frac{k_{G}}{m_{G}}}$, and the quality factor of the gyroscope is $Q_{G}=\frac{m_{G\;}\omega_{G}}{c_{G}}$. Therefore, the simplified forms of equations of motion for the vibrating ring gyroscope are presented as Equations (45) and (46), respectively.

(45) $$\ddot{G_{1}}+\frac{\omega_{G}}{Q_{G}}\dot{G_{1}}+{\omega_{G}}^{2}G_{1=}\frac{F_{1}}{m_{G}}$$

(46) $$\ddot{G_{2}}+\frac{\omega_{G}}{Q_{G}}\dot{G_{2}}+{\omega_{G}}^{2}G_{2=}\frac{F_{2}}{m_{G}}$$

### 2.3. Implication of Resonance Analysis on MEMS Vibrating Ring Gyroscope Design

**Figure 8:**
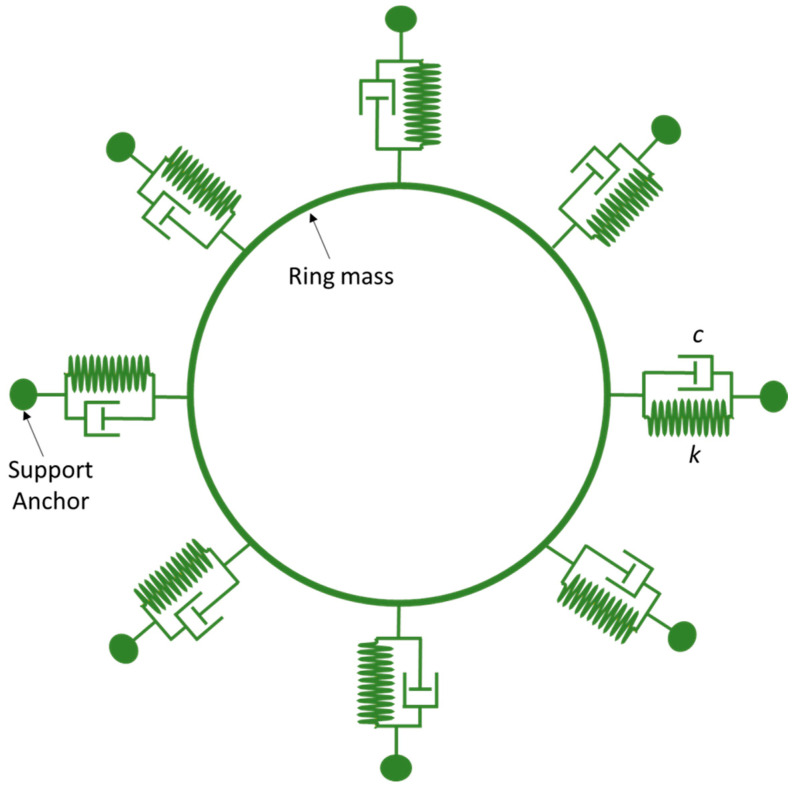
A spring mass damping system for a MEMS vibrating internal ring gyroscope.

In this section, we will investigate the resonance of elliptical modes of vibrations for the MEMS vibrating ring gyroscope. The spring-mass system for the MEMS vibrating internal ring gyroscope is shown in Figure 8. The gyroscope system comprises the internal vibrating ring mass, spring damper, and externally supported anchors. There are eight support beams attached to the internal vibrating ring mass, and the whole vibrating structure is connected to the externally placed supporting anchors.

**Figure 9:**
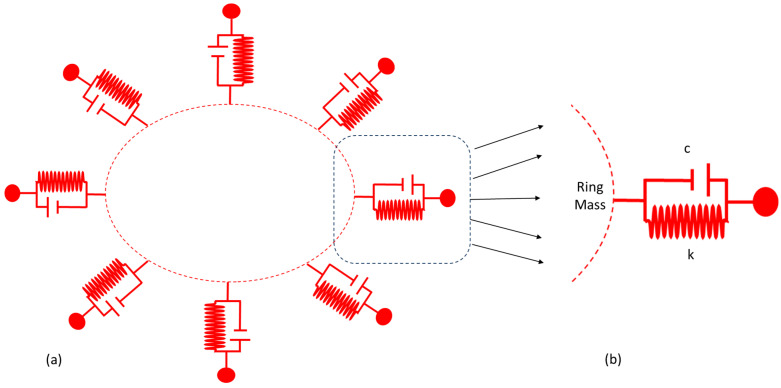
The driving mode of a spring mass system for a vibrating ring gyroscope. (**a**) The complete architecture. (**b**) The single ring resonator is shown as highlighted trough arrows.

In the driving operation, the MEMS vibrating ring gyroscope comprises a resonator with a single degree of freedom. To understand the driving mechanism of the MEMS vibrating ring gyroscope’s dynamics, it is recommended to consider the one-eighth portion of the ring gyroscope, as shown in Figure 9b. The one-eighth portion comprises a single resonator with one degree of freedom with one support beam and damping connected to the external anchor. The same concept applies to the sensing mechanism, too, as the vibrating ring gyroscope comprises a symmetric design.

Let us consider Figure 9b; it has a single resonator with one degree of freedom. As previously derived, Equation (27) is implemented on the one-eighth portion of the vibrating ring gyroscope.

(47) $$m_{G}\ddot{G}+c_{G}\dot{G}+k_{G}G=F$$

In a further analysis, the natural frequency ${”\omega }_{N}”$ is a parameter that analyzes the oscillation behavior of the vibrating structure without damping. The damping factor “ζ” measures the damping present in the vibrating system relative to critical damping. The equation of the motion for the one-eighth portion becomes

(48) $$\ddot{G}+2\;\zeta \omega_{N}\dot{G}+{\omega_{N}}^{2}G=\frac{F}{m_{G}}$$

The Laplace transform function for the single degree of freedom is considered to further simplify Equation (48).

(49) $$\frac{G\;(s)}{F\;(s)}=\frac{1}{ms^{2}+2\;\zeta \omega_{N}s+{\omega_{N}}^{2}}$$

When a vibrating ring resonator is subjected to the harmonic force $F=F_{0}\;sin(\omega t)$ at the given frequency “ω”, the system responds with a steady-state harmonic excitation that can be expressed as Equation (50).

(50) $$G=G_{0}\sin(\omega t+\vartheta )$$

Here, ${"G}_{0}”$ is the response amplitude and can be determined as

(51) $$G_{0}=\frac{F_{0}}{\sqrt{k\left[{\left(1-{\left(\frac{\omega }{\omega_{N}}\right)}^{2}\right)}^{2}+{\left(2\zeta \frac{\omega }{\omega_{N}}\right)}^{2}\right]}}$$

(52) $$\vartheta =-\tan^{-1}\left[\frac{2\zeta \omega }{\omega_{N}}\times \frac{1}{1-{\left(\frac{\omega }{\omega_{N}}\right)}^{2}}\right]$$

Here, $F_{0}$ is the amplitude of the applied force, ω is the frequency of the applied force, $\omega_{N}$ is the natural frequency of the vibrating ring, and ϑ is the phase angle between the applied force and the response.

Now, the vibrating ring gyroscope is considered as a resonator with two degrees of freedom comprising driving and sensing axes. As discussed earlier, the equations of motion for the two-degrees-of-freedom resonator for the vibrating ring gyroscope are presented as Equations (53) and (54), respectively. The vibrating ring gyroscope oscillates in one direction when there is no rotation applied to the device.

(53) $$\ddot{G_{1}}+2\;\zeta_{1}\omega_{1}\dot{G_{1}}+{\omega_{1}}^{2}G_{1}=F_{1}\;sin(\omega t)$$

(54) $$\ddot{G_{2}}+2\;\zeta_{2}\omega_{2}\dot{G_{2}}+{\omega_{2}}^{2}G_{2}=0$$

In the given situation, when no external force is applied to the ring gyroscope’s secondary motion, the primary response $G_{1}$ and secondary response $G_{2}$ of the gyroscope after the closed form solution can be written as Equations (55) and (56), respectively.

(55) $$\begin{array}{cc} G_{1}\left(t\right)=A_{1}e^{{-\zeta }_{1}t} & \sin\left(t\omega_{1}\sqrt{1-{\zeta_{1}}^{2}}+\vartheta_{1}\right)\\ & +\frac{F_{0}}{\sqrt{{\left({\omega_{1}}^{2}-\omega^{2}\right)}^{2}+4{\zeta_{1}}^{2}{\omega_{1}}^{2}\omega^{2}}}\left[\sin\left(\omega t+\tau \right)\right] \end{array}$$

(56) $$G_{2}\left(t\right)=0$$

The phase shift “τ” due to the primary oscillation motion can be determined as

(57) $$\tau =\tan^{-1}\left(\frac{2\zeta_{1}\omega_{1}\omega }{{\omega_{1}}^{2}-\omega^{2}}\right)$$

After the natural oscillations are considered damp, the solutions of Equations (55) and (57) reveal that the frame of the sensitive element, in conjunction with the proof mass, performs forced oscillations along the driving axis with an amplitude corresponding to the excitation force. During this time, the proof mass will not shift away from the driving axis, and therefore, the sensing output will be zero.

Previously, we considered that there is no rotation when the vibrating ring gyroscope oscillates only in the driving axis. This shows that the oscillation will remain absent in the sensing axis. Now, we analyze the behavior of the vibrating ring gyroscope when it rotates with a constant angular rate. The rotation will bring cross-couplings $c_{1}$ and $c_{2}$ in both axes. Therefore, we have effective accelerations $a_{1}$ and $a_{2}$ due to the external forces applied to the system.

(58) $$\ddot{G_{1}}+2\;\zeta_{1}\omega_{1}\dot{G_{1}}+\left({\omega_{1}}^{2}-\Omega^{2}\right)G_{1}=a_{1}-2c_{1}\Omega \dot{G_{2}}$$

(59) $$\ddot{G_{2}}+2\;\zeta_{2}\omega_{2}\dot{G_{2}}+\left({\omega_{2}}^{2}-\Omega^{2}\right)G_{2}=a_{2}+2c_{2}\Omega \dot{G_{1}}$$

If there are no forces affecting the oscillations in the sensing axis, then $a_{2}=0$, and we will assume there is no coupling between the driving and sensing axes. Hence, sensing oscillation will only work on the angular rate.

The solutions of Equations (58) and (59) are of interest due to their role in measuring the angular rate, while the natural solution pertains to transient phenomena. If the excitation of the driving oscillation follows a harmonic pattern, then the acceleration resulting from the excitation forces can be expressed as Equation (60).

(60) $$a_{1}=\left|a_{10}\right|\cos\left(\omega t+\vartheta \right)$$

Here, $a_{1}$ is the effective acceleration, $a_{10}$ is the amplitude of the effective acceleration, ω is the excitation frequency, and ϑ is the phase constant. The solutions for the driving and sensing oscillations with respect to the decoupling frame are expressed as Equations (61) and (62), respectively.

(61) $$G_{1}\left(t\right)=\left|A_{1}\right|\cos\left(\omega t+\vartheta_{1}\right)$$

(62) $$G_{2}\left(t\right)=\left|A_{2}\right|\cos\left(\omega t+\vartheta_{2}\right)$$

The complex amplitudes related to the gyroscope system are defining the forced oscillation at the given frequencies $A_{1}$ and $A_{2}$.

(63) $$A_{1}=A_{10}e^{i\vartheta_{10}}$$

(64) $$A_{2}=A_{20}e^{i\vartheta_{20}}$$

Now, we have constant amplitudes $A_{10}$ and $A_{20}$ and phases $\vartheta_{10}$ and $\vartheta_{20}$ for diving and sensing oscillations. The above solutions will be substituted in Equations (58) and (59).

(65) $$\left({\omega_{1}}^{2}-\Omega^{2}-\omega^{2}+2\zeta_{1}{i\omega }_{1}\omega \right)A_{1}+a_{1}i\omega \Omega A_{2}=a_{10}$$

(66) $$\left({\omega_{2}}^{2}-\Omega^{2}-\omega^{2}+2\zeta_{2}{i\omega }_{2}\omega \right)A_{2}-a_{2}i\omega \Omega A_{1}=0$$

The equations to determine the solutions of the complex amplitudes are given below.

(67) $$A_{1}=\frac{a_{10}\left({\omega_{2}}^{2}-\Omega^{2}-\omega^{2}+2\zeta_{2}{i\omega }_{2}\omega \right)}{\Delta }$$

(68) $$A_{2}=\frac{2c_{1}a_{10}i\omega \Omega }{\Delta }$$

(69) $$\begin{array}{c} \Delta ={(\omega_{1}}^{2}-\Omega^{2}-\omega^{2})\;{{(\omega }_{2}}^{2}-\Omega^{2}-\omega^{2})-(4c_{1}c_{2}\omega^{2}\Omega^{2}+4\zeta_{1}\zeta_{2}\omega_{1}\omega_{2}\omega^{2})\\ \;\;\;\;\;\;\;+2i\omega \left[\zeta_{1}\omega_{1}\left({{(\omega }_{2}}^{2}-\Omega^{2}-\omega^{2}\right)+\zeta_{2}\omega_{2}({{(\omega }_{2}}^{2}-\Omega^{2}-\omega^{2})\right] \end{array}$$

The phases $\vartheta_{1}$ and $\vartheta_{2}$ of the driving and sensing oscillations that correspond to the angles of $A_{1}$ and $A_{2}$, can be determined by the given equations.

(70) $$\tan\left(\vartheta_{1}\right)=\frac{2\omega \left[\left[\zeta_{1}\omega_{1}\left({{(\omega }_{2}}^{2}-\Omega^{2}-\omega^{2}\right)+\zeta_{2}\omega_{2}\left({{(\omega }_{1}}^{2}-\Omega^{2}-\omega^{2}\right)\right]\left({{(\omega }_{2}}^{2}-\Omega^{2}-\omega^{2}\right)+\omega_{2}\zeta_{2}\right]}{{{(\omega }_{2}}^{2}-\Omega^{2}-\omega^{2})\left[{(\omega_{1}}^{2}-\Omega^{2}-\omega^{2}){{(\omega }_{2}}^{2}-\Omega^{2}-\omega^{2})-\omega^{2}\Omega^{2}\left(c_{1}c_{2}+4\zeta_{1}\zeta_{2}\omega_{1}\omega_{2}\right)\right]-4\zeta_{2}\omega_{2}\omega^{2}}$$

(71) $$\tan\left(\vartheta_{2}\right)=\frac{\left[{(\omega_{1}}^{2}-\Omega^{2}-\omega^{2}){{(\omega }_{2}}^{2}-\Omega^{2}-\omega^{2})-\omega^{2}\Omega^{2}\left(c_{1}c_{2}+4\zeta_{1}\zeta_{2}\omega_{1}\omega_{2}\right)\right]}{2\omega \left[\zeta_{1}\omega_{1}\left({{(\omega }_{2}}^{2}-\Omega^{2}-\omega^{2}\right)+\zeta_{2}\omega_{2}\left({{(\omega }_{1}}^{2}-\Omega^{2}-\omega^{2}\right)\right]}$$

### 2.4. Mode Matching in Vibrating Ring Gyroscopes

The vibrating ring gyroscope operates on two primary modes of oscillations. The first is driving oscillations, where the ring continuously vibrates in two orthogonal axes like an elliptical ring. The second vibration mode is sensing oscillations, which is when the device experiences rotation, where the vibrating ring gyroscope oscillates at 45 degrees between two orthogonal axes and maintains the same elliptical vibration mode [34,35,36,37,38].

**Figure 10:**
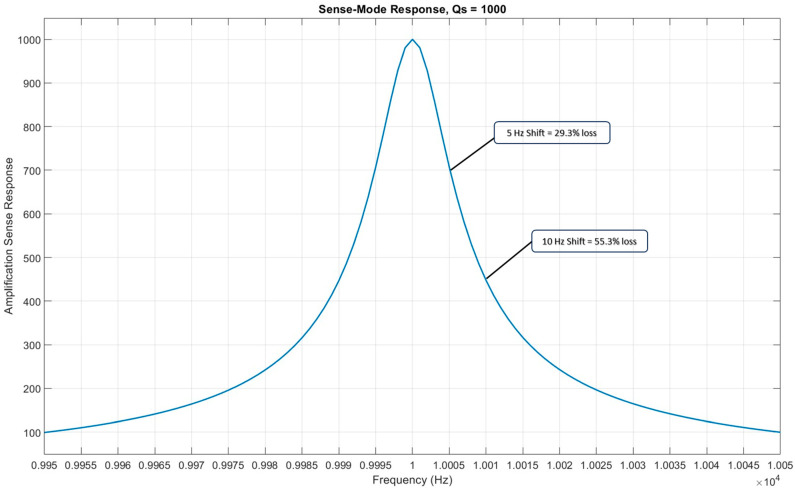
The effect of a resonance frequency shift on the sense mode amplification.

In an ideal situation, the driving and sensing resonance frequencies should be matched. This mode matching amplifies the sensing mode response to the angular rate input, making the gyroscope performance more accurate and precise. We consider a case where the vibrating ring gyroscope sensing frequency $\omega_{S}$ is 10 kHz and has a quality factor $Q_{f}$ of 1000. At perfect mode matching, the amplification factor of the response is 1000, which is the same as the quality factor. The presented scenario is shown in Figure 10. A vibrating ring gyroscope operates at or close to its resonant frequency and is more susceptible to any fluctuations in the system that shift the resonance frequency. As shown in Figure 10, a 5 Hz deviation can cause a significant 29% loss in the amplification factor. A 10 Hz shift increases to a 55% loss in the amplification factor.

**Figure 11:**
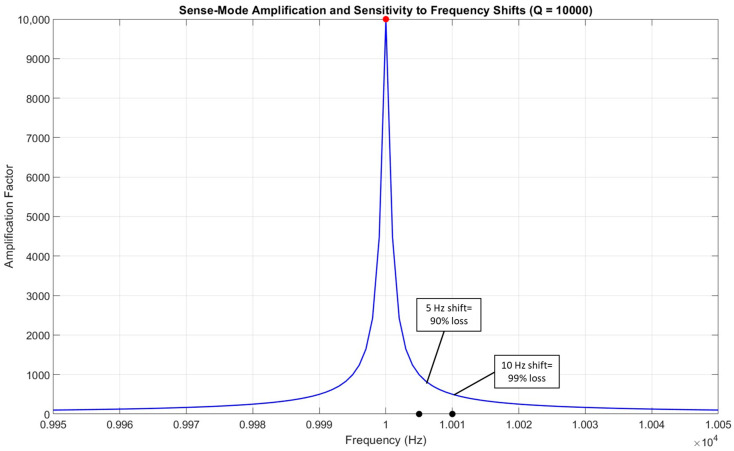
A high-quality factor gyroscope is more susceptible to a high loss of gain, black dots indicate 5 Hz and 10 Shift, while red dot shows a peak value of the resonance.

In another scenario, if the quality factor increases to 10,000, which gives us a higher amplification factor, it provides better results. This also narrows down the operational bandwidth. In this case, if the frequency just shifts by 5 Hz, it will decrease the gain to 90%, as shown in Figure 11.

As discussed earlier, operating mode-matched resonance frequencies is quite challenging due to many factors, like unwanted vibrations, microfabrication tolerances, and environmental factors. Therefore, operating the gyroscope in the sensing frequency with low peaks is better as it will not impact the sensing amplification factor much. This will deliberately offset the sensing resonance frequency with a small percentage of the driving resonance frequency. This intentional gap between the driving and sensing resonance frequencies is typically known as a frequency separation system.

Most MEMSs’ vibrating gyroscopes work on the principle of translational motion of a single-proof mass system. Translational motion provides better gyroscopic performance and simple microfabrication processes because of a single-proof mass system. Some of the gyroscopes utilize the rotational motion of the spring-mass system but add some complex operational processes. On the other hand, dual mass-spring system gyroscopes require more complex microfabrication processes, which sometimes contribute to unwanted errors in gyroscope operations. The intentional gap between two operating resonance frequencies is presented as Equation (72).

(72) $$∆f=f_{S}-f_{D}$$

## 3. Conclusions

We presented a thorough investigation of the in-plane vibrational dynamics of the MEMS internal vibrating ring gyroscope with semicircular beams. The analysis presented a detailed analytical model of the vibrating ring gyroscope. This included elliptical vibrational modes assuming inextensional displacements, resulting in detailed equations that could effectively demonstrate a vibrating ring resonator’s radial and tangential displacement. The symmetric design structure of the vibrating ring gyroscope provided identical pairs of elliptical vibrational modes. The importance of mode matching on the performance of the gyroscope was presented, showing that there is a disadvantage when the sensing frequency has the same peak value as the driving frequency. The derived motion equations are important in order to optimize the design of the vibrating ring gyroscope for increased performance and higher sensitivity.

## Data Availability

The original contributions presented in the study are included in the article, further inquiries can be directed to the corresponding authors.
